# Quo Vadis translational neuroscience?

**DOI:** 10.1515/tnsci-2025-0393

**Published:** 2026-02-26

**Authors:** Ekrem Dere

**Affiliations:** Department of Behavioral and Clinical Neuroscience, Ruhr-University Bochum, Bochum, Germany; Sorbonne Université. Institut de Biologie Paris-Seine, Unité de Formation et de Recherche des Sciences de la Vie (UFR 927), Paris, France

**Keywords:** nanocarrier-based drug delivery, neurologic diseases, psychiatric diseases, pharmacological treatment, brain repair and regeneration, animal models of human diseases

## Abstract

Translational neuroscience is a research discipline that aims to transfer findings from basic research in neuroscience into clinical applications. The main goal of this research discipline is to gain molecular and mechanistic insight into brain diseases and to devise novel diagnostic tools and therapeutic applications. This review is organized in three major sections which address recent developments in diagnostic innovation, therapeutic translation and integrative modelling. Furthermore, the most urgent problems and challenges of translational neuroscience as a research discipline are presented and viable solutions are discussed. Promising novel methods are presented, and suggestions for new research approaches are made. Although translational neuroscience deals with diseases of the most complex human organ that there is, the brain, it is likely to turn out to be one of the few disciplines in life sciences that will continue to see steady progress and discoveries.

## Introduction

### What is translational neuroscience?

The field of translational neuroscience is so broad that any attempt to formulate a definition must remain inadequate. Nevertheless, if one wanted to try a very general one, it would be something like this: Translational neuroscience is a research discipline, which has developed from the larger field of neurosciences, that attempts to transfer findings from basic research in neuroscience into clinical applications for diseases of the brain. If this translation is successful, new hypotheses about the pathogenesis of brain diseases can emerge, molecular and mechanistic disease models can be further developed, diagnostic tools, treatment monitoring, and clinical care can be refined, and finally more efficient and better tolerated therapeutic applications can be devised and implemented. Translational research that aims to bridge the gap between basic research, disease models and clinical practice, requires an interdisciplinary approach and the integration of knowledge and methods from a wide range of different disciplines, including neurogenetics, neurobiology, neuroanatomy, neurophysiology, neurology, psychology, and psychiatry.

## Diagnostic innovation

One key task within the field of translational neuroscience is the continuous improvement of available diagnostic tools and the development of novel tools for disease risk assessment, prodromal stage detection, disease diagnosis and subtype specification, detection of disease trajectories, progression acceleration and deceleration, detection of early signs of relapse, the determination of appropriate prevention strategies, and the measurement of treatment success. Diagnostic innovation in the future might be based on findings from basic, preclinical, and clinical research that aims to determine genetic and epigenetic risk factors [1], [2], [3], [4], [5], [6], uses polygenetic and epigenetic risk scores to develop disease risk prediction models, tries to identify protective factors that might counteract disease manifestation [7], [8], [9], and searches for diagnostic biomarkers in the blood or cerebrospinal fluid [10], [11], [12], [13].

### Genetic biomarkers: how much further is it?

The fact that broad research efforts regarding the genetic basis of mental, psychiatric, or neurological disorders have so far been used only to a limited extent for diagnostic purposes is due to the complexity of these disorders. The significance of genetic studies for the diagnosis of mental, psychiatric, or neurological disorders is limited by the fact that these disorders are subject to a complex genetic architecture [14]. Disease-associated genetic variants are never causal, i.e., necessary and sufficient for the development of a disease. Frequently, disease-associated genes merely modulate the risk of disease manifestation, and non-genetic environmental factors (e.g., via epigenetic mechanisms) play an important role in the development of, for example, mental and psychiatric disorders [15], 16]. It has also been proven that adverse environmental factors, in the presence of genetic risk factors, can exacerbate the development of these disorders [17]. Genetic and non-genetic factors can trigger disease manifestation, and the relative weight of these factors in disease development varies between diagnoses. Another factor that complicates the translation of basic research findings into diagnostic practice is the fact that genetic influences or risk genes exhibit a very prominent overlap across various disease diagnoses [18], [19], [20], [21]. It must also be considered that psychopathological disease categories or diagnoses often lack a specific correspondence at the neurobiological or genetic level and tend to manifest themselves on an in-between levels as neurodivergence or endophenotypes. In summary, regarding the genetic basis of mental, psychiatric, and neurological disorders, genome-wide association studies can identify common genetic variants that may increase disease risk, but individual risk genes or allele variants only increase the risk minimally, depending on the literature consulted by approximately 1–1.5 times. Therefore, polygenic risk scores might be more promising for the assessment of genetic risks. However, even polygenetic risk scores cannot predict disease manifestation with sufficient accuracy and reliability, suggesting that the path to genetic biomarkers that can be used as a diagnostic tools is still very long and stony. The unfulfilled hopes of the search for crucial risk genes have already led some experts to speak of the post-GWAS era [22], 23]. The most promising way to develop reliable biomarkers for determining disease risk seems to be a combination of different methods and approaches including polygenetic risk scores, transcriptome and proteome analysis (to control for epigenetic modulation of gene silencing) and the determination of environmental risk factors.

### Endophenotypes as joints between genetic risk and disease

Research into the genetic basis of mental, psychiatric, or neurological disorders has not been able to identify single risk genes or allele variants that are not stand-alone and causative triggers for diseases. They are not even decisive for the induction of individual symptoms of a disease syndrome.

Mental disorders can be viewed as a combination of various neurodivergent processes or endophenotypes. Many of the identified genetic risk factors may influence brain development, thereby increasing vulnerability to mental, psychiatric, and neurological disorders [24]. The adverse influence of risk genes on brain development can manifest as a single endophenotype or a combination of endophenotypes (neurodivergent manifestations) that have a closer relationship to the neurobiological causes or pathophysiological mechanisms of the disease than the clinical phenotype or individual symptoms of the disease [25]. Endophenotypes are therefore manifestations of systemic in-between levels that act as mediators between the effect of the risk gene at the neuronal network level and the psychopathological experience and behavior that manifests as a disease symptom [26], 27]. It appears that endophenotypic in-between levels are more directly influenced by susceptibility or disease risk genes than the psychopathological experience and behavior at the syndrome level on which the diagnosis is based [28]. Endophenotypes can thus be considered an expression of brain vulnerability, which can (also) be triggered by disease-associated gene allele variants and is likely to play a critical role in the development of diseases [29], 30]. However, it must also be noted that endophenotypes, in themselves, cannot be used for disease diagnosis because they are episode- or state-independent markers that, while they may co-occur more frequently with a disease, can also be found in healthy individuals, partly because they are often quantitative or continuously distributed deviations from the neurotypical norm. Therefore, endophenotypes by themselves cannot be used as biomarkers for mental, psychiatric, and neurological disorders. However, the predictive validity of disease risk determined via polygenic risk scores could potentially be improved by incorporating endophenotype measurements.

### Further challenges in diagnostic innovation

A major obstacle in the transfer of research results from basic research into clinical practice is the fact that specific neurological or psychiatric diseases are not yet well understood in their breadth of diversity, i.e., different neuropathology, disease progression, symptoms, and comorbidities. It often seems as if many different causes lead to the supposedly same disease respectively diagnose, and that individual patients often exhibit very different non-overlapping disease factors.

### Clinical studies

Another major problem in this context are false positive and negative diagnoses in clinical phase 2 studies, which lead to a heterogeneous clinical test population in which the efficiency of translational therapeutic approaches is to be tested [31], [32], [33], [34]. To address this problem, the Research Domain Criteria approach was developed in which patients with neurological and psychiatric symptoms are not just labeled with diagnoses (syndromes with distinct core symptoms) but are assessed using a series of individual continuous symptom scales in order to develop a symptom combination oriented individual therapy program, that can benefit from translational therapeutic approaches [35], 36].

### Translational neuroscience and the replication crisis

The replication crisis has severely impacted the translational neuroscience research domain and the life sciences as a whole, leading to a range of procedures designed to improve the quality, validity, and reproducibility of published data [37], 38]. The safety measures implemented by the scientific community to enhance the reproducibility of scientific research and prevent scientific misconduct include the open science concept for making published datasets available, the publication of study protocols (see ref. [39] for an example for a randomized clinical trial protocol), and preregistration before data collection begins [40]. In particular, preregistration of the study design, including directional hypotheses, detailed methods, information on participant recruitment, power analyses and group sizes, evaluation procedures, and statistical analysis, has been established as the gold standard for preclinical and clinical research. There are now even journals dedicated to publishing randomized controlled trials in the health sciences (see ref. [41] for a recent exemplary publication in a clinical trials journal), and even publications with guidelines that must be followed if the final publication of the collected datasets deviates from the procedures described in the preregistration, or if researchers are required to justify such deviations [42]. A very positive view of the measures taken to overcome the replication crisis and a hopeful outlook for the future were recently published by Korbmacher and colleagues [43] under the heading “credibility revolution”. For a more critical evaluation of the “credibility revolution” see the interesting ideas and views of E. D. Klonsky [44].

Many of the implemented safety and control measures are indeed suitable for increasing the quality and reproducibility of published data and have certainly strengthened awareness of good scientific practice and vice versa, scientific misconduct. However, there is a risk that the credibility revolution could contribute to an impoverishment of methodological breadth and hinder the development of new methods and research approaches [45]. The courage to undertake high-risk or exploratory studies without very detailed hypotheses could, due to the strong preregistration “pressure” or “publication barrier” imposed, lead to the “anxious” and conservative choice of well-established, classical, and semi- or fully automated methods, thus resulting in methodological impoverishment or, if things turn really bad, even systematic errors (f. e. if the commonly used methods are inadequate). This could hinder scientific progress in the long run. For example, in psychological research on episodic memory, methods were used for a long time that didn’t even measure the core features of these memory contents. These methods were only modified under the “unconventional” pressure from animal studies, leading to a common standard for animal and human experimental research in this area [46], [47], [48].

Even before the replication crisis came up and was widely debated (which most severely affected research in psychological research), there were inconsistent findings, and a statement or postulated relationship was only believed if it could be confirmed using many different methods and approaches, or if it wasn’t regularly refuted. In other words, if many researchers around the world arrive at the same basic conclusion using sometimes very different methods, this conclusion can be believed just as readily as if hundreds of studies using the same method consistently replicated the same result. It must also be considered that attempts to replicate findings through maximum standardization have not always been successful [49] and many groundbreaking (if not all) discoveries were more or less due to chance. A very prominent example is the discovery of rewarding intracranial self-stimulation by Olds and Milner [50], who actually intended to investigate something entirely different. Even before the replication crisis, there were guidelines from scientific teaching on empirical experimental design, methodology, and biostatistics regarding how data must be collected, analyzed, and interpreted, and that scientific integrity is the highest good of a scientist who tries to understand nature and not the other way around, imposing their own laws upon it. Transparency and replicability must not replace scientific curiosity and innovation, and it must not be forgotten that what distinguishes good scientists from excellent ones lies primarily in their ability to recognize and formulate problems, to find methods to investigate the problem, and ultimately to recognize an important connection, even and especially when it doesn’t fit the established pattern.

## Therapeutic translation

### Current status of therapeutic translation of findings from basic research

Prominent examples for the implementation of the translational neuroscience approach are the exploration of new psychopharmacological targets [51], [52], [53], [54], [55], [56], [57], [58], cell gene therapy [59], [60], [61], [62] or the modification of disease-related epigenetic changes in neurological and psychiatric diseases [63], [64], [65]. Furthermore, methods have been developed including non-invasive brain stimulation, such as transcranial magnetic gene stimulation [66] and transcranial direct current stimulation [67], which are thought to modulate neuroplasticity by inducing changes in cortical excitability and connectivity that might repair and restore dysfunctional neuronal network functions [68]. Yet other approaches include deep brain stimulation [69], 70] as well as brain-machine interfaces [71] that can support and facilitate recovery from psychiatric symptoms and neurorehabilitation after nervous system injury or degeneration.

### Psychopharmacology: dose finding and non-responders

It is known that not all patients respond to psychopharmacological treatment in the same way and to the same extend. Some patients are even classified as treatment-resistant and are referred to as non-responders [72], 73]. The individual response to standard doses can also differ in terms of intensity and duration of medication effects, as well as with regard to side effects. Treatment resistance is generally defined as having no or only a slight reduction in symptoms at therapeutic doses [74]. Psychiatric and neurological clinical practice often encounters problems in determining the appropriate individual dose within a therapeutic reference range. For example, therapeutically necessary concentrations of the medication in the blood plasma might not be achieved, making significant symptom reduction without serious side effects or toxicity impossible. One possible explanation for these inter-individual differences in drug responsiveness is that non-responders may metabolize the drug so effectively that the necessary therapeutic blood plasma concentrations within the safe dosage range are not reached. Cytochrome P enzymes are among the enzymes used in the body to break down endogenous and extraneous substances into simpler molecules. CYP450 enzymes exhibit a broad range of enzymatic activity together with relatively low substrate specificity, meaning that many different substances are metabolized. CYP450 enzymes are ubiquitously distributed and are found in the liver, kidneys, small intestine, lungs, and central nervous system. They are involved in the biotransformation of substances (also known as catabolism) and play an important role in the defense against extraneous substances (xenobiotics, pesticides, chlorinated solvents, etc.). In the liver, CYP450 enzymes, for example, oxidize water-insoluble substances to prepare them for detoxification and excretion. CYP450 enzymes also play an important role in drug metabolism and, among other things, determine the elimination half-life of drugs [75], 76].

At the genetic level 20 CYP450 gene families with a total of 57 CYP450 genes have been identified so far. Furthermore, an extensive genetic polymorphism has been identified for CYP450 genes. Approximately 12 CYP450 enzymes are involved in human drug metabolism. Among the CYP450 genes, CYP450 alleles with increased activity, reduced activity, or even complete loss of function have been found [77], [78], [79].

In the case of complete loss of function of CYP450 genes, the drug might accumulate in the body. Detoxification does not occur, and the consequences might be intensified effects, severe side effects, or even toxic effects already at standard doses. Genetic studies have identified gene duplications, or deletions, as well as allele variants with increased or reduced activity, Regarding drug metabolism, theoretically at least four different phenotypes can be distinguished: 1. Ultra-rapid metabolizers with an accelerated metabolism, for example, due to CYP450 genes duplication with overexpansion and an insufficient standard dose; 2. Average metabolizers with “normal” CYP450 gene alleles; 3. Slow metabolizers with a reduced metabolism and CYP450 genes defect resulting in partial or complete loss of function; and 4. Ultra-slow metabolizers with a severely reduced metabolism due to a CYP450 gene defects with complete loss of function. In the latter case, the standard dose is far too high, and severe side effects and toxicity can occur. Given the importance of the CYP450 enzymes for drug metabolism, it would be interesting to further pursue the analysis of CYP450 gene variants to identify non-responders or treatment-resistant patients in clinical settings and to be able to predict the individual response to psychotropic drugs. This could avoid or shorten lengthy dose finding processes, the trial and error of different medications in monotherapy, numerous drug switches, or risky augmentation attempts [80], 81].

### Nanocarrier-based drug delivery to the brain as a promising translational research field

The development of novel pharmacological treatment approaches for neurological and psychiatric disorders, is frequently thrown back because of the poor blood-brain barrier permeability of the experimental substances [82], 83]. In general, it is a great challenge to achieve sufficient concentrations of drugs in brain regions that are affected by neuropathological processes or injuries, e.g., brain regions in which one or more neurotransmitter systems are dysfunctional or inflammatory processes are prevalent. Fortunately, nanocarrier-based drug delivery systems have been recently developed to transport drugs more effectively across the blood-brain barrier to create a better bioavailability of the drugs together with lower systemic toxicity [84], [85], [86], [87], [88].

Nanocarriers are colloidal particles that can be as small as 1 nm (up to 100 nm), are typically composed of polymeric nanoparticles, dendrimers, or lipids, which enables the encapsulation of therapeutic agents and drugs in the size of small molecules, proteins, hydrophobic drugs, and nucleic acids. Substances enclosed in these nanocarrier capsules are protected from premature enzymatic degradation and ensure drug stability, bioavailability, better regulated release kinetics and biocompatibility [84], [85], [86], [87], [88].

Through modifications of the surface structure of these nanoparticles with the incorporation of various functional moieties, including polyethylene glycol or specific antibodies, the circulation time of the encapsulated drugs might be enhanced and targeted and drug delivery to specific tissues or cells would be within the realm of possibility. The first successful steps towards targeted drug delivery have already been undertaken [89]. The surface structure of nanocarriers can be modified to recognize structures that express transferrin receptors and thus guide the nanocarriers to amyloid plaque deposits to release substances such as naringenin that can counteract neurotoxicity in these regions [90], [91], [92]. Another exciting approach is the development of conditional stimuli-responsive drug release by nanocarriers, which release the encapsulated drug only in response to microenvironmental triggers including extracellular pH, temperature, or specific enzymatic activity [93], [94], [95]. This allows the drug to be released under certain conditions or pathological states. The nanocarrier approach can be further optimized to reduce the risk of side-effects, (e.g., immune responses against the nanocarriers) using computational modelling to determine optimal nanocarrier dimensions, surface structure, and drug-load, as well as to predict the pharmacokinetic activity including spatial distribution patterns and peak concentrations.

Research into the treatment of mental, psychiatric, and neurological conditions (e.g., neurodegenerative diseases such as Alzheimer’s and Parkinson’s) with nanocarrier systems is currently still in the preclinical or early clinical phase. A search for “neurodegenerative diseases” and “nanomedicine,” “nanocarriers,” or “nanoparticles” in the clinical trials database “ClinicalTrials.gov” yielded only about a dozen results. Even fewer studies were found for psychiatric conditions such as schizophrenia (2 results) and major depression (0 results). Clinical trials outside the field of translational neuroscience have used, among other things, polymeric nanoparticles, quantum dots, liposomes, and solid-lipid nanoparticles. So far, the success of some of these applications has been limited due to issues of safety, biocompatibility, and scalability [77], 78], 96], 97]. However, refined nanocarrier systems based on lipid nanoparticles, of which some are already approved for clinical use, would be particularly interesting for transporting substances to target structures in the brain, because they would not only allow for a significant reduction in the administered doses of the substances and perhaps their application over longer periods, but also because, compared to other delivery vehicles, they would elicit fewer immunogenic reactions due to higher biocompatibility [77], 78]. For an overview of the ethical, regulatory, safety, and translational challenges of brain-penetrating nanocarrier systems, see a recent publication by Saraswathi and colleagues [98].

### The use of artificial intelligence in the design of nanocarrier-based drug delivery systems

The application of deep machine learning applications such as generative artificial intelligence based on adversarial networks in the design of disease-specific nanomaterials respectively nanocarriers holds exciting potential. In this context machine learning and generative adversarial networks can help to analyze extensive datasets on material properties, biophysiological interactions, and patient data to determine the optimal structural and pharmacokinetic and physicochemical properties for targeted, safe and efficient drug delivery [99], [100], [101], [102], [103], [104].

#### Machine learning for data integration

Possible ways to solve the problems that are associated with a multi-factorial experimental approach are offered by new technologies such as artificial intelligence [105], which can identify and interpret higher-order interactions, big data statistical methods (beyond Principal component analysis [106] and discriminant cluster analysis [107] can handle hundreds of thousands of medical records [108], and repositories for clinical and basic research data [109]). Machine learning approaches are particularly important for complex, multifactorial diseases with unclear pathogenesis that are based on higher-order interactions between genetic, epigenetic, and environmental influences. In this context, for example, an integrative analysis of signaling and metabolic pathways, immune infiltration patterns, together with analyses of the genome, epigenome, transcriptome, and proteome could be helpful in constructing more valid etiological and diagnostic models (see ref. [110] for an exemplary case using major depression). Another interesting area of ​​application is in the field of drug repositioning, which aims to create new applications for already approved drugs as a cost- and time-efficient alternative to *de novo* drug development [87], 88], 111]. Interesting applications of machine learning for data integration can also be found in the prediction of the generalizability of clinical psychotropic drug efficacy studies, for example on the effectiveness of antidepressants [112].

#### Generative artificial intelligence for drug response prediction and hypothesis building

One potential application of generative artificial intelligence is the prediction of the effectiveness of psychopharmacological medication, for example, the antipsychotic efficacy of new substances in patients with psychotic disorders or schizophrenia. Recently, Yee and colleagues, for example, trained a machine learning-based classifier to categorize plasma levels of inflammatory markers in schizophrenia for the prediction of antipsychotic responsiveness. Using this method, the authors were able to classify patients as either antipsychotic-responsive, clozapine-responsive, or clozapine-resistant [113].

The analysis of large sets of patient data including genomic data for the assessment of the individual genetic risk allele burden, and individual metabolic characteristics (e.g. cytochrome-P450-enzyme alleles), in addition to medical records, and demographic information can help to predict how an individual might respond to a particular nanocarrier formulation in order to optimize personalized treatment dosing schedules [114].

Increasingly powerful computers and quantum technology [115], [116], [117], [118], [119] might be able to generate a pool of virtual test partners and patients, as well as virtual experimental settings and paradigms for the development and testing of new therapeutic approaches [120], [121], [122], [123].

### Stimulation of endogenous brain repair mechanisms and neuroplasticity

There is a great deal of evidence suggesting that the administration of neurotrophic factors can support neurogenesis, neuronal survival, axonal growth and remyelination after brain injury [124], [125], [126], [127]. Neurogenesis stimulating agents and stem cell-based grafting treatments have been used for the repopulation of areas with neuron and glial cell loss [60]. However, these methods struggle with severe technical problems such as stem cell survival, correct integration into neuronal networks, and the risk of cell proliferation and tumor formation. Therefore, an important challenge for translational research is to find efficient, controlled, and safe ways to activate endogenous repair mechanisms, neurogenesis, neuroplasticity, and the reinstatement of the extracellular matrix in the injured brain to induce regeneration of damaged components of the brain. The use of nanocarrier-based delivery of therapeutic agents to the affected brain areas and these brain areas only would be of inestimable value.

## Integrative modelling

There is still a urgent need to improve currently available disease models for translational research to ensure future breakthroughs (of which there have unfortunately not been too many in the last 3 decades). In what follows, the existing shortcomings of preclinical and clinical models will be discussed, the problems of the currently prevailing reductionist research approach will be debated, and proposals will be made for a new research approach that could build on new technological possibilities.

### Preclinical research: issues associated with current animal models

But even before the clinical phase 2 and 3 stages, problems exist with the disease models used [128], 129]. Animal models of human diseases are required to have important levels of construct, face, and predictive validity [130], [131], [132]. Unfortunately, these validity criteria are rarely met satisfactorily. A lack of predictive validity has hampered the development of effective psychotropic drugs [54], 55], 133], 134], although more optimistic assessments were also presented [135]. Predictive validity refers to a basic condition in which an already approved drug reduces disease-like symptoms in an animal model just as effectively as in the patient, so that this animal model can be used to evaluate new substances and preparations in preclinical studies. Unfortunately, it must be stated that animal models of neurologic or psychiatric diseases frequently fail to predict novel drug efficacy at a reasonable scale [134], 136]. Still worse, animal models for neurological and psychiatric disorders are often inadequate or incomplete in terms of face validity, i.e., the ability to reproduce and measure the symptoms observed in patients [137], 138]. A particularly prominent example are animal models for schizophrenia. Although encouraging approaches have been proposed [139], 140], to date, the cardinal positive symptoms of schizophrenia, namely delusions and hallucinations, have not been convincingly modeled in animals. Likewise, there are a number of diseases whose main characteristics, for example mania and cyclic mood changes as in bipolar disorder, are difficult to reproduce or simulate in animal models [141], although some encouraging progress has been made recently [142].

Insufficient or missing animal models for preclinical research also affect translational research in disorders of consciousness [143]. Although changes in consciousness are a frequent symptom in neurological and neuropsychiatric diseases, pre-clinical and clinical research into this phenomena seems to be quite rare, because of serious conceptual problems in the definition of consciousness and the lack of validated paradigms for measuring states of consciousness (beyond extreme and basic states such as coma, anesthesia, sleep and wakefulness) in human experiments and animal models, although some progress has been made recently [144], [145], [146], [147], [148].

### Construct validity issues

Adequate construct validity, i.e., the quality with which the causes of the brain disease or its neuropathology are reproduced in animal models, is also not always satisfactory. This is particularly true for genetic animal models of neurological and psychiatric disorders [149], [150], [151]. Gene therapies which targeting specific genes might be a valid approach for some monogenic disorders [152], but they fall short in brain diseases with polygenetic disease mediators [153], [154], [155].

To complicate matters further, even in cases where animal models of complex diseases appear acceptable in terms of their construct validity, the behavioral symptoms and disease progression may differ significantly from the patient’s condition. A good example of high construct validity together with moderate face validity is the 5xFAD mouse model of Alzheimer’s disease. In 5xFAD mice five familial Alzheimer’s disease mutations (APP KM670/671NL, APP I716V, APP V717I, PSEN1 M146L, PSEN1 L286V) are overexpressed in neurons of the forebrain. This genetic burden leads to stable beta-amyloid-related pathology, including brain inflammation, microgliosis, synaptic and neuronal loss [156]. At ages where the brain pathology is strongly progressed in 5xFAD mice, learning and memory deficits are either still absent or developing as compared to the wild-type mice [156], [157], [158], [159], [160]. These examples underline the necessity to invent novel and refine existing *in vivo* animal models of human brain disease to assess the potential of novel therapies at a pre-clinical stage.

### The reductionist pitfall

Up to date, the success of the translation of basic research into clinical practice was smaller than it probably could have been. This is due to the fact that neurological and psychiatric diseases are usually multifactorial phenomena. Translational applications in the clinical field normally begin with a discovery from basic research using a reductionist *in vitro* or *in vivo* approach using a model system or patient material. This means that a finding at a microscopic level must be translated to the macroscopic multi-faceted level of a disease. A major obstacle to the successful implementation of findings from basic research into clinical practice stems from a fundamental principle of experimental research [161], [162], [163].

In the classic definition of an experiment, the influence of an independent variable (with n values) on one or more dependent variables is investigated. Ideally, experimental partners, test animals, tissue or cell material are randomly assigned to the experimental and control groups. To investigate multi-factorial neurological or psychiatric diseases in which, for example genetic risk alleles interact with adverse environmental factors to trigger a disease, one would have to conduct more complex experiments with several simultaneously manipulated independent variables already at the basic research level. However, limits of statistical processing and analysis as well as the interpretability of complex findings and interactions between independent variables are quickly reached with such a multi-factorial approach (but see ref. [164], 165]). To make progress at this point, the classical reductionist approach would have to be supplemented by a multi-factorial experimental approach, to develop a research approach that takes the true complexity of brain diseases into account.

### From reductionism to multi-factorial basic research

At present, it must unfortunately be stated that there is still no curative treatment for neurological, neurodegenerative, and psychiatric diseases. Patients usually must deal with poor prognosis, must expect progressive disability and frequently also a reduced life expectancy. Up to know it is thought that the key to the understanding and treatment of complex neurological and psychiatric diseases is the dissection of the disease into small measurable and manipulable pieces of defects and dysfunctions (comparable to a puzzle) that can be examined, studied and repaired in isolation. If a treatment is found for all individual defects and dysfunctions, then the disease can be treated at least symptomatically, and its progression can be slowed down. Unfortunately, this approach was not satisfactory in terms of therapeutic success. Even though research in the field of translational neuroscience has expanded our understanding of disease causes and mechanisms, it has contributed only little to the development and successful implementation of efficient therapies [166]. The decisive step in the translation process therefore remains the greatest challenge. As mentioned above, this is due to the multifactorial etiology of brain diseases, but also to the heterogeneity of the affected patients. To address this problem, basic research must integrate the sheer volume of individual findings and re-evaluate them using a multifactorial approach, shifting from a reductionist to a multifactorial research approach. The translational research approach must be redirected from dissecting complex diseases into small symptom fractions and mediators to the simulation of disease complexity.

Understanding complex neurological and psychiatric disorders requires basic research that examines how neural systems respond to the manipulation of multiple variables that induce multifactorial interactions or higher-order interactions. Where are the nodes where higher-order interactions have the greatest impact?

Most effective therapeutic approaches for treating brain diseases can only alleviate symptoms and delay disease progression for a certain period but not halt the progression of the disease or reverse it. Research prospects for curing brain diseases have yet to be developed. The peak of these positive developments occurred some time ago, followed by major drawbacks, and there have been no major breakthroughs for a long time. Leading the field out of stagnation requires, firstly, a complementation of reductionist approaches with multifactorial studies and the use of new high throughput technologies in the areas of artificial intelligence and quantum computer science.

## Conclusion and future perspectives

In summary, the field of translational neuroscience will likely achieve its greatest progress and breakthroughs if research focuses on the following areas of scientific potential and growth: 1. The integrated analysis and processing of “multi-omics” datasets (that is genetic, epigenetic, transcriptomic, and proteomic data). 2. The establishment of standardized translational biomarkers and improved diagnosis and classification systems for CNS-related diseases. 3. The development of human-disease-relevant brain organoid models to test for *in vivo* drug activity. 4. The further development of nanocarrier-based drug delivery systems for mental, psychiatric, and neurological disorders. 5. The development of virtual brain, patient, and clinical trial systems that would allow to test virtual patient groups in virtual clinical trials.

As part of all efforts to advance translational research methodology, ethical, societal, and equity dimensions of translational neuroscience, including data sharing, gender equity, patient neuro- and gender diversity, as well as the equal and barrier-free access to emerging therapies, must be given highest priority. An overview of these important topics can be found in recent publications by Fang et al. [167]; Manzini et al. [168]; Smith et al. [169]; and Veras et al. [170].

Considering what has already been achieved and with respect to encouraging new developments in the field, it is fair to say that the field of translational neuroscience will remain one of the few life science disciplines that will continue to see groundbreaking discoveries and breakthroughs. However, translational neuroscience is also one of the disciplines that deals with the most complex organ in the human body. However, the developments described above give hope for a bright future. The continued interdisciplinary efforts of scientists from various disciplines will lead to improved treatments for neurological and psychiatric disorders in the further course of the 21st century.
